# Characteristics of The Bleached Microbiome of The Generalist Coral *Pocillopora damicornis* from Two Distinct Reef Habitats

**DOI:** 10.1093/iob/obad012

**Published:** 2023-04-28

**Authors:** J L Bergman, F Ricci, W Leggat, T D Ainsworth

**Affiliations:** Biological, Earth, and Environmental Sciences, University of New South Wales, Sydney, NSW, 2052,Australia; Biological, Earth, and Environmental Sciences, University of New South Wales, Sydney, NSW, 2052,Australia; School of BioSciences, University of Melbourne, Melbourne, VIC, 3010Australia; School of Environmental and Life Sciences, University of Newcastle, Callaghan, NSW, 2308,Australia; Biological, Earth, and Environmental Sciences, University of New South Wales, Sydney, NSW, 2052,Australia

## Abstract

Generalist coral species may play an important role in predicting, managing, and responding to the growing coral reef crisis as sea surface temperatures are rising and reef wide bleaching events are becoming more common. Pocilloporids are amongst the most widely distributed and studied of generalist corals, characterized by a broad geographic distribution, phenotypic plasticity, and tolerance of sub-optimal conditions for coral recruitment and survival. Emerging research indicates that microbial communities associated with Pocilloporid corals may be contributing to their persistence on coral reefs impacted by thermal stress; however, we lack detailed information on shifts in the coral–bacterial symbiosis during bleaching events across many of the reef habitats these corals are found. Here, we characterized the bacterial communities of healthy and bleached *Pocillopora damicornis* corals during the bleaching events that occurred during the austral summer of 2020 on Heron Island, on the southern Great Barrier Reef, and the austral summer of 2019 on Lord Howe Island, the most southerly coral reef in Australia. Regardless of reef location, significant differences in α and β diversities, core bacterial community, and inferred functional profile of the bleached microbiome of *P. damicornis* were not detected. Consistent with previous reports, patterns in the Pocilloporid coral microbiome, including no increase in pathogenic taxa or evidence of dysbiosis, are conserved during bleaching responses. We hypothesize that the resilience of holobiont interactions may aid the Pocilloporids to survive Symbiodiniaceae loss and contribute to the success of Pocilloporids.

## Introduction

Anthropogenically induced climate change is driving record warm temperatures and rates of warming culminating in large-scale bleaching and mortality events across the world's coral reefs (Hughes et al. 2017; van Woesik and Kratochwill 2022). Bleaching events are becoming increasingly frequent and severe (Hughes et al. 2017, 2018; Eakin et al. 2019; Sully et al. 2019). For example, on Australia's Great Barrier Reef (GBR), anomalously warm temperatures have triggered six mass coral bleaching events since the late 1990’s, four of which have occurred since 2016 (2016, 2017, 2020, and 2022; and GBRMPA 2017, GBRMPA, AIMS, & CSIRO Reef Snapshot 2022). Mean coral cover in the central section of the GBR was also affected by the repeated bleaching events in 2016 and 2017, declining from 22% coverage in 2016 to 14% coverage in 2018 (AIMS 2018), but increasing to 36% in 2022 (AIMS 2022). Bleaching events have also impacted high-latitude reefs over the last two decades, including at Sodwana Bay, South Africa (Celliers and Schleyer 2002), Lord Howe Island (LHI), Australia (Dalton et al. 2011, 2020; Harrison et al. 2011), Rottnest Island, Australia (Thomson et al. 2011), the Houtman Abrolhos Islands, Australia (Abdo et al. 2012; Smale et al. 2012), and Norfolk Island, Australia (Parks Australia Report 2021). Thus, the consequences of ocean warming are evident on coral reefs worldwide, including those located at high latitude reefs that have been hypothesized as global warming refuges (Kim et al. 2019).

Studies of coral bleaching induced mortality events are increasingly assessing changes in the composition and functional role of the microbial communities of the corals that undergo a loss of symbiotic algal partners (Symbiodiniaceae) due to bleaching partial mortality and colony mortality (Cziesielski et al. 2019; Gardner et al. 2019; van Oppen and Blackall 2019). It is clear from this research that changes in community composition in response to bleaching are not consistent across corals, reef locations or bleaching events. Studies have shown that the composition of the bacterial microbiome associated with corals can either change (Mouchka et al. 2010; Tout et al. 2015; McDevitt-Irwin et al. 2017) or remain unchanged during a thermal stress event (Tracy et al. 2015; Hadaidi et al. 2017; Epstein et al. 2019; van Oppen and Blackall 2019; Bergman et al. 2021). In some studies, thermal stress has been shown to correlate with shifts in the microbial community through an increase of opportunistic pathogens (*Acropora millepora* [Bourne et al. 2008] and *Acropora, Porites*, and *Pocillopora* [Maher et al. 2019]) and that pathogens found in the communities of heat-stressed corals resemble those found in diseased corals (*Porites compressa* [Thurber et al. 2009] and *P. damicornis* [Tout et al. 2015]). Similarly, some bleached corals have been shown to host higher proportions of *Vibrio* and *Acidobacteria* than healthy corals (Mouchka et al. 2010; Morrow et al. 2018). Furthermore, the “Anna Karenina Principle” (AKP) of dysbiosis suggests that bacterial microbiome changes induced by perturbations are stochastic and lead to unstable community states instead of a specific microbiome configuration associated with stress (Zaneveld et al. 2017). However, many studies are also increasingly pointing to stability or resilience of the microbiome while the host corals are undergoing Symbiodiniaceae loss, bleaching, and/or thermal stress. For example, in many studies of Pocilloporid corals, the microbiome has been shown to remain stable throughout thermal stress (*P.* *acuta* [Epstein et al. 2019], *P. damicornis* [Brener-Raffalli et al. 2018; Bergman et al. 2021], and the *P. damicornis* species complex [Ziegler et al. 2017; Pogoreutz et al. 2018]). Stability of the microbiome is defined here as no significant increase in diversity of bacterial communities of the host coral during heat stress, such as that observed during two degree heating weeks of thermal stress in *P. acuta* (Epstein et al. 2019). While it has been suggested that a stable microbiome may contribute to resilience against dynamic environmental conditions (Ziegler et al. 2017; Grottoli et al. 2018), alternative hypotheses suggest this may also prevent the colonization of beneficial microbes (Reshef et al. 2006) and contribute to a breakdown of the meta-organism relationship among host, symbionts, and microbiome (Tracy et al. 2015). The diversity of documented coral microbial community responses to thermal stress also suggests that the response of the coral microbiome to thermal stress will vary under certain degrees of stress, extent of bleaching, or past history of bleaching. Clearly, the coral holobiont and microbial community responses are complex and therefore likely vary across the substantial diversity of Scleractinia, coral reef habitats, and the severity of heat stress and bleaching impacts to the coral host.

One of the most broadly distributed families of corals are the Pocilloporids, a widespread taxonomic group of generalist scleractinian corals. Generalist coral species thrive across a breadth of environmental conditions and are generally considered more tolerant of sub-optimal conditions than species existing within a narrow environmental niche (Richmond et al. 2005; Clavel et al. 2011; Darling et al. 2012). The Pocilloporids include *P. damicornis*, shown to be one of the most geographically cosmopolitan and generalist species of scleractinian coral in the pan Pacific, which has also often been characterized as sensitive to bleaching (Dalton et al.; Loya et al. 2001; van Woesik et al. 2011; Claar and Baum 2019). However, generalists are regarded to be more successful than specialists in the current climate crisis due to extensive plasticity and tolerance of sub-optimal environmental conditions (Clavel et al. 2011; Chichorro et al. 2019). *P. damicornis* has been characterized as bleaching-sensitive on the GBR, despite containing higher density of Symbiodiniaceae than more bleaching-resistant species (Ulstrup et al. 2006); however, its ability to rely on heterotrophic feeding may contribute to its persistence in a changing environment whilst undergoing bleaching responses (Todd 2008; Schmidt-Roach et al. 2014). The cosmopolitan distribution of *P. damicornis*, as well as its potential for success in changing environmental conditions as a generalist, make it an ideal study species for examining trends in coral physiology and stress response in a range of environmental conditions.

**Fig. 1:**
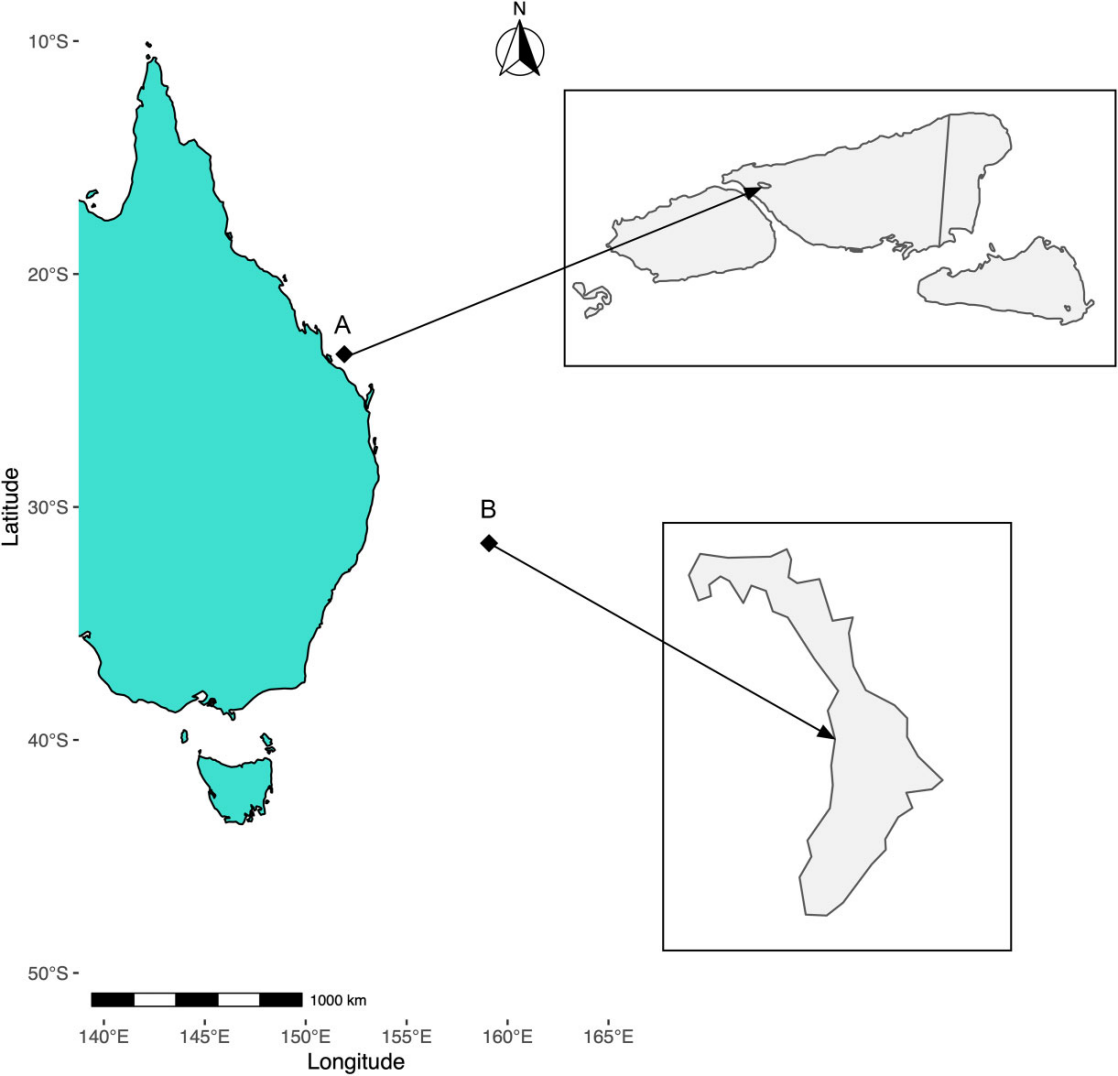
Conceptual map showing study site locations. (**A**) HI and (**B**) LHI.

Here, we therefore investigated the microbiome of the generalist coral species *P. damicornis* during reef wide coral bleaching events that occurred in 2019 and 2020 on a subtropical and tropical shallow coral reef lagoonal ecosystem (Fig. 1). *P. damicornis* colonies were sampled during the onset of reef wide bleaching on the tropical reef lagoons of Heron Island (HI) on the southern GBR in summer of 2020 and LHI in summer of 2019. This study characterizes the microbiome composition and inferred bacterial functions of *P. damicornis* during the bleaching events on these reefs to investigate the characteristics of a bleached coral microbiome for the generalist coral species of two different coral reef habitats.

## Methods

### Sample collection and DNA extraction

*Heron Island* (*HI*). Single branch fragments per colony (∼4 cm) of *P. damicornis* were collected on snorkel using needle-nose pliers sterilized between samples with 70% molecular-grade ethanol from the shallow reef flat at 1–2 m depth in the research zone of HI in March 2020. Samples were transported to Heron Island Research Station in individual WhirlPak© bags. Bleaching of *P. damicornis* colonies was recorded during ecological monitoring of the reef wide bleaching event (Ainsworth et al. 2021) and colonies were selected for sampling based on visual signs of bleaching (white, *n* = 5) and stability with algal symbionts (e.g., no clear visual indication of bleaching stress, herein referred to as healthy, *n* = 5). Samples were collected from the center of colonies >3 m apart and separated by distinct sand patches to reduce the likelihood that colonies were clonal (Permit: G19/41974.1). Samples were immediately snap-frozen in WhirlPak© bags dipped in liquid nitrogen and stored at −80°C until processing for DNA extraction following the protocol outlined below. Each entire coral fragment, including the coral skeleton, (1.34 ± 0.14 g, mean ± SE, *n* = 10) was added to a 2 mL tube containing 1.4 mm ceramic spheres (Matrix E, MP Biomedicals). DNA extraction followed manufacturer protocols using a QIAGEN QIAmp DNA Mini Kit (cat. #56304) with minor adjustments as briefly outlined here. Digestion buffers were doubled for all protocols, a step which greatly increased DNA yields following overnight digestion. A FastPrep-24 5G homogenizer (MP Biomedicals, Irvine, CA, USA) was programed to run three rounds of 20 s each (6.0 m/s) to homogenize the sample. Following homogenization, all samples were incubated overnight (18–24 hours) at 56°C. Samples were then centrifuged (3 min at 6000 x *g*) to pellet calcium carbonate remaining from the coral skeleton. The resulting supernatant was transferred to a new 2 mL tube, centrifuged again to pellet out any remaining calcium carbonate (3 min at 20,000 x *g*), and supernatant was transferred to a new 2 mL tube. Following the remainder of the manufacturer's protocol, AL buffer and 100% molecular-grade ethanol were doubled to increase in proportion to the increased ATL and Proteinase K buffer volume prior to vortexing and adding to the collection column (QIAmp DNA Mini Kit, Qiagen). The manufacturer's protocol was followed for the remainder of the procedure and DNA eluted to 60 µL.

*Lord Howe Island* (*LHI*). Single branch fragments per colony (∼4 cm) of *P. damicornis* were collected on snorkel using needle-nose pliers sterilized between samples with 70% molecular-grade ethanol from the shallow fringing Lagoon reef at 1–2 m depth on the western side of LHI in March 2019 (Permit MEAA19/206). Bleached (*n* = 5) and healthy (*n* = 5) colonies were selected based on the visual health surveys outlined in Steinberg et al. (2022) during the reef wide bleaching event in March 2019. Sterile 16% paraformaldehyde ampules (Electron Microscopy Sciences, cat # 50980487) were used for the preparation of 4% paraformaldehyde (PFA) by a 1:3 dilution with phosphate buffered saline (PBS) solution (PBS tablets [Invitrogen, Waltham, MA, USA]) in UltraPure DNA/RNA-Free Distilled Water (ThermoFisher Scientific, Waltham, MA, USA). Samples were then added to 50 mL conical tubes immediately following collection and covered with the 4% PFA preservative solution. After 14 hours, PFA solution was removed and replaced with the DNA/RNA free PBS for storage. Samples were stored at 4°C for ∼3–6 months. DNA was extracted from coral fragments collected from LHI (1.29 ± 0.16 g, mean ± SE, *n* = 10) by adding each entire fragment to a 2 mL tube containing 1.4 mm ceramic spheres (Matrix E, MP Biomedicals). DNA extraction followed manufacturer protocols using a RecoverAll™ Total Nucleic Acid Isolation Kit for FFPE (cat #AM1975) protocol with minor adjustments as briefly outlined here. 400 µL of digestion buffer and 8 µL of Proteinase K were added prior to bead-beating as described previously. Following homogenization, all samples were incubated overnight (18–24 hours) at 50°C and centrifuged twice (3 min at 6000 x *g* and 3 min at 20,000 x *g*) to pellet and remove calcium carbonate remaining from the coral skeleton. Isolation additive and 100% molecular-grade ethanol were doubled to increase in proportion to the increased digestion buffer and Proteinase K buffer volume prior to vortexing and adding to the collection column. The manufacturer's protocol was followed for the remainder of the procedure and DNA eluted to 60 µL.

*16S rRNA gene amplicon sequencing and analysis.* For all samples, extracted DNA concentration and purity were quantified using a Qubit Fluorometer and Qubit dsDNA broad-spectrum assay kits (Life Technologies, Thornton, NSW, Australia). Extracted DNA was stored at −20°C prior to PCR amplification and sequencing. DNA extraction, amplification, and sequencing were performed on all samples as well as on two negative controls (no sample template, to account for contamination introduced during DNA extraction) prepared per site (*n* = 20 samples plus four negative controls in total). Sequencing was performed by MR DNA (Molecular Research LP, Shallowater, TX, USA) on the Illumina MiSeq platform following manufacturer's guidelines. The 16S rRNA gene V1–V3 regions PCR primers 27F/519R, commonly used in studies of the coral microbiome (e.g., Glasl et al. 2019; Marchioro et al. 2020) were used in a 35 cycle PCR using the HotStarTaq Plus Master Mix Kit (Qiagen, Germantown, MD, USA) under the following conditions: 95°C for 5 minutes, followed by 35 cycles of 95°C for 30 seconds, 53°C for 40 seconds, and 72°C for 1 minute, after which a final elongation step at 72°C for 10 minutes was performed. Samples were multiplexed using unique dual indices, pooled together in equal proportions based on molecular weight and DNA concentrations, and purified using calibrated Ampure XP beads. The pooled and purified PCR product was then used to prepare an Illumina DNA library and sequenced on the Illumina MiSeq (2 × 300 bp-paired end reads) following the manufacturer's guidelines.

### Data analysis

Sequence data were analyzed using Quantitative Insights Into Microbial Ecology version 2 (QIIME2, (Bolyen et al. 2019). After denoising and primer removal using the DADA2 pipeline (Callahan et al. 2016), taxonomy was assigned to amplicon sequence variants (ASVs) in QIIME2 using a naïve Bayes classifier trained on SILVA 138 reference sequence and taxonomy files pre-formatted for use with QIIME2 using RESCRIPt (Robeson et al. 2021). ASVs assigned as “chloroplasts,” “mitochondria,” or unassigned (classification absent at a phylum level) were removed and excluded from the final ASV table. Contaminant removal was conducted in R based on contaminants identified in two negative controls per site/extraction protocol (version 4.1.2) using the package decontam at a threshold of 0.5, which implements a statistical classification procedure that identifies contaminants in sequencing data (Davis et al. 2018). Data analysis was conducted in R using the packages *phyloseq* (McMurdie and Holmes 2013) and *ampvis2* (Andersen et al. 2018). Linear discriminant analysis effect size tests (LEfSe, [Segata et al. 2011]) were conducted using the package *microbiomeMarker* (Cao 2020) on unrarefied data, using an LDA cutoff of 1. At a higher LDA cutoff, no differences were observed in bleached samples. The functional composition of bacterial communities was inferred using both software platforms PICRUSt2 (Douglas et al. 2020) and Tax4Fun2 (Wemheuer et al. 2020), and overlapping KEGG Orthologs (KO) between PICRUSt2 and Tax4Fun2 outputs were used for final data visualization with PICRUSt2 abundance values. The coral core microbiome was determined for species-specific community taxa at a prevalence threshold of 30, 60, and 90% using the “occupancy method” defined by Custer et al. (2023), e.g., present in equal to or greater than X% of samples (Ainsworth et al. 2015; Hernandez-Agreda et al. 2018a; Ricci et al. 2022).

### Statistical analysis

All statistical analyses were performed in R, using the package *vegan* (Dixon 2003) for multivariate statistics and *ggplot2* (Wickham and Chang 2016) for data visualization. Alpha diversity metrics were analyzed using separate one-way ANOVAs for each treatment (healthy and bleached) using unrarefied and rarefied data (Shannon, Chao1, and Inverse Simpson). In cases where residuals were not normally distributed (determined via Shapiro test), the non-parametric Kruskall–Wallis test was used to analyze differences in alpha diversity metrics between healthy and bleached corals at each location. For beta diversity, Euclidean distance matrices on centered log-ratio (clr)transformed data were analyzed between healthy and bleached corals at each location. Homogeneity of dispersion around group centroids was assessed for each beta diversity metric between bleached and healthy corals using PERMDISP (*betadisper* function in vegan). A permutational multivariate analysis of variance (PERMANOVA, *n* = 9999, *adonis* function in vegan) was performed to test for dissimilarities in microbial community composition between healthy and bleached samples.

Prior to assessing the beta diversity of healthy and bleached corals at each site, we evaluated the effectiveness of different normalization methods on our dataset. A common challenge in interpreting data from 16S rRNA ecological datasets is finding a normalization technique that best fits data characteristics. As 16S rRNA data is compositional, the starting point for analysis used a ratio transformation of the data (Gloor and Reid 2016; Gloor et al. 2017). A common problem with ASV tables is the high degree of zero inflation of the proportional data (up to 90%) (Paulson et al. 2013). Following the recommendations of Gloor et al. (2017), *a* + 1 count was added to the ASV tables and we used a clr transformation prior to undertaking community composition analysis (as used by Gloor et al. 2017). The benefit of a clr transformation (Aitchison 1982) is that ratio transformations can capture the relationships between features in the dataset regardless of if the data are counts or proportions. clr-transformed values are scale-invariant, which means that the same ratio will be obtained from samples with high or low read counts. Given the low read counts within coral microbiome samples (due to high DNA concentration of host and dinoflagellate DNA in the mixed holobiont sample), we also compared a clr transformation and Aitchison distance matrix to the widely used method of data rarefaction. For DNA amplified coral samples from HI to rarefaction was at a depth of 733 (90% of the minimum sequence count) resulting in 633 taxa identified across the 10 samples, removing 41% of taxa. It is important to note that for DNA amplified from coral samples from LHI rarefaction was at a depth of 8352 (90% of the minimum sequence count) resulting in 326 taxa across 10 samples, removing ∼10% of taxa. We therefore assessed community composition using the clr-transformed data and the Aitchison distance matrix, as no taxa were lost using this method.

## Results

*Sequencing statistics.* In total, 311,748 sequences from 20 samples and 4 negative controls were generated within the study. Quality control and removal of chloroplasts, mitochondria, unassigned ASVs (classification absent at a phylum level), and potential contaminants resulted in the retention of 304,678 sequences with a mean of 15,233 ± 2,437 (± SE) reads per sample, ranging from a minimum of 815 reads to a maximum of 41,209 reads (Table S1). Clustering at ASV level and removing negative controls yielded 1427 distinct ASVs for analysis of the microbial community. 1085 ASVs were identified in coral samples from HI (*n* = 10 samples, 815–40,635 reads per sample), and 365 ASVs were identified in coral samples from LHI (*n* = 10 samples, 9280–33,218 reads per sample).

### HI coral bacterial communities

**Fig. 2:**
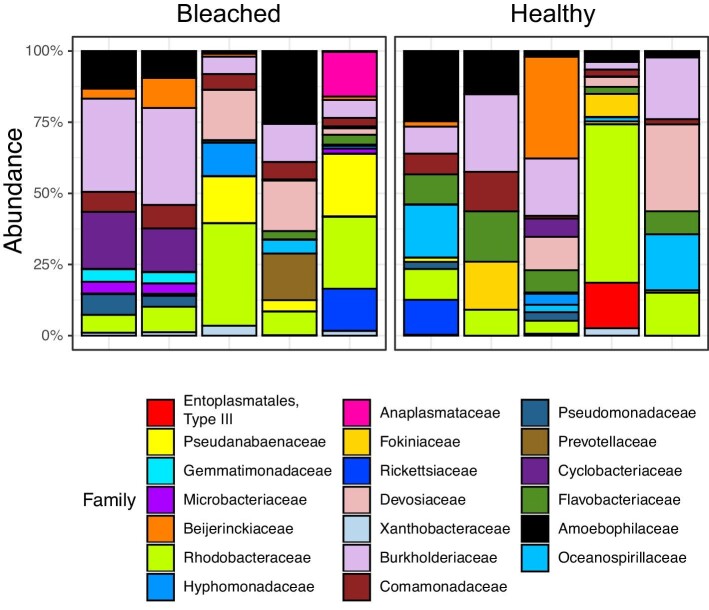
Relative % abundance of ASVs in the HI community microbiome, agglomerated down to top 20 families and sorted by health status.

*Characteristics of the bleached coral microbiome from HI.* Phylum-level assignment of bacteria ASVs indicated the dominance of Proteobacteria in both healthy (83% of taxa) and bleached (64% of taxa) samples. Family-level assignment of bacterial ASVs indicated the dominance of Rhodobacteraceae (15% of taxa) in healthy samples from HI, followed by Sphingomonadaceae (5% of taxa) and Colwelliaceae (4% of taxa). However, the families “Type III,” Burkholderiaceae, and Amoebophilaceae had the highest average relative abundances in healthy samples (Fig. 2). Rhodobacteraceae (19% of taxa) was also the most prevalent family in bleached HI samples, followed by Pseudanabaenaceae (5% of taxa) and Colwelliacae (4% of taxa). The families with the highest relative abundance in bleached HI samples were Burkholderiaceae, Anaplasmataceae, and Amoebophilaceae (Fig. 2), similar to the community observed from the control samples.

**Fig. 3:**
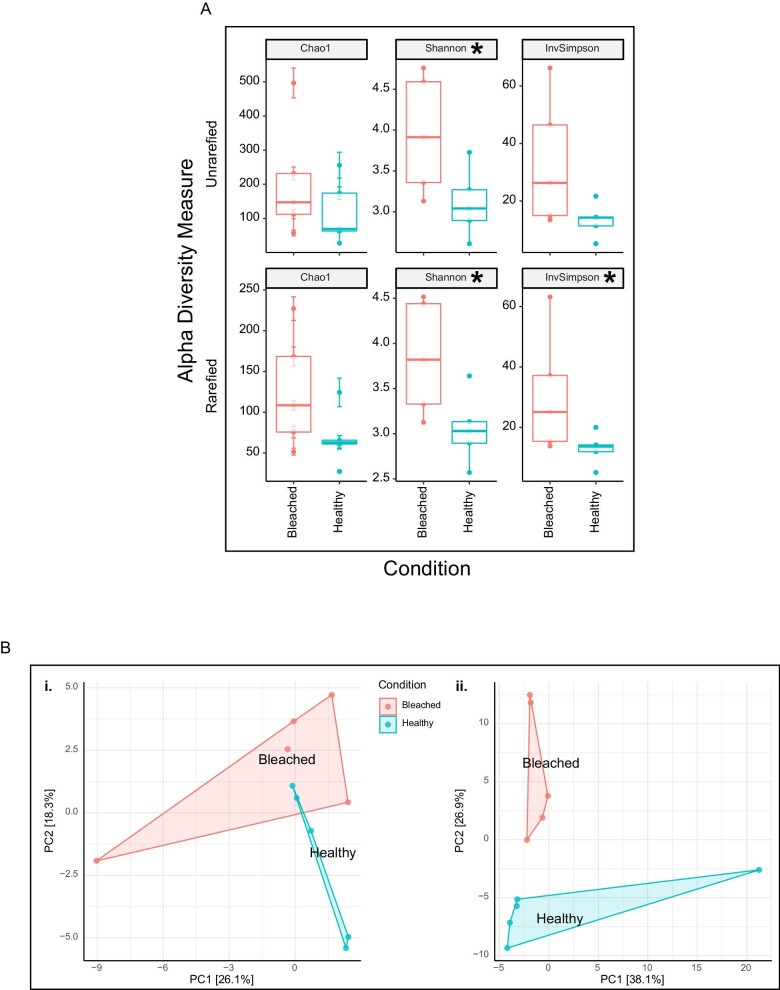
Community-level analyses for HI. (**A**) Alpha diversity metrics on both unrarefied and rarefied data comparing healthy and bleached samples. Significant differences are indicated by an *. (**B**) Principal component analyses (PCoA) comparing clr-transformed (1) and rarefied (2) Euclidean distance matrices. No significant differences were observed in beta diversity metrics.

*Beta diversity did not differ between bleached or healthy bacterial communities from HI, but Shannon diversity did.* Shannon diversity differed between healthy and bleached corals (χ^2^ = 3.938, *p* = 0.047), but there was no statistical difference between healthy and bleached communities from HI samples in ASV richness (Chao1, χ^2^ = 0.534, *p* = 0.465) and dominance (Simpson, χ^2^ = 3.153, *p* = 0.076) (Fig. 3A). Beta diversity did not differ between healthy or bleached bacterial communities of corals from HI during the 2020 bleaching event (PERMANOVA on clr-transformed Euclidean distances, *F* = 1.195, *p* = 0.093; Fig. 3B).

**Fig. 4:**
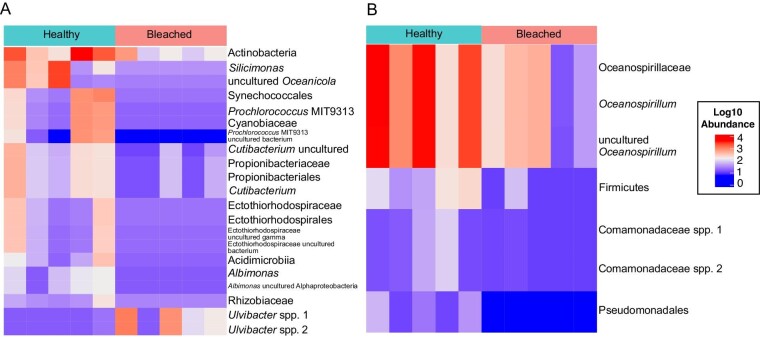
Heatmap resulting from LEfSe analysis, showing differentially abundant ASVs in bleached and healthy corals for (**A**) HI and (**B**) LHI. Abundances were log(10) transformed for visualization. Taxa are listed to the lowest level of classification identified.

*Differentially abundant ASVs in bleached and healthy coral samples from HI do not include pathogenic taxa.* Employing linear discriminant analysis effect size (LeFSe) analysis, we identified members of the coral microbiome at an ASV level that were differentially abundant in bleached or healthy samples. In corals from HI, *Ulvibacter* spp. were found in higher relative abundance in healthy coral tissues (Fig. 4A). More taxa were found to be higher in relative abundance in bleached corals than healthy, with individual ASVs from the families Rhodobacteraceae, Cyanobiaceae, Propionibacteriaceae, Ectothiorhodospiraceae, and Rhizobiaceae found at a higher abundance in bleached samples (Fig. 4A). However, potentially disease-associated taxa (e.g *Vibrio*) were not found at a higher abundance in bleached coral samples than in healthy coral samples from HI (Fig. 4A). In healthy coral samples, an uncultured *Vibrio* ASV was present in one sample at 0.15% relative abundance. In bleached coral samples, one *V. neocaledonicus* ASV was present in one sample at 0.01% relative abundance. There was also no differential abundance of *Endozoicomonas* ASVs in either healthy or thermally stressed samples, a common bacterial associate in Pocilloporid corals (Pogoreutz et al. 2018; Epstein et al. 2019; Voolstra and Ziegler 2020; Ricci et al. 2022). There was a very low relative abundances of *Endozoicomonas* ASVs in one healthy sample (0.11%) and none detected in bleached samples.

**Fig. 5:**
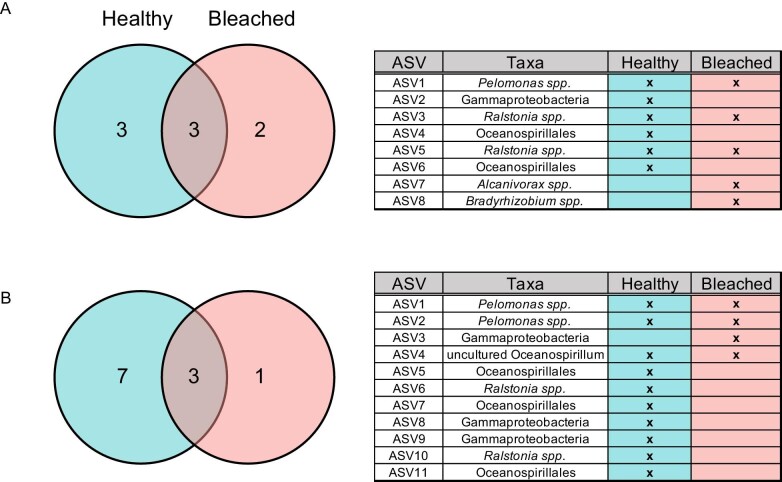
Venn diagrams showing common bacterial phylotypes shared between bleached and healthy *P. damicornis* colonies at 90% prevalence at (**A**) HI and (**B**) LHI. Venn diagrams are accompanied by a table listing core ASVs at the lowest level of classification identified.

*Coral core microbiome in corals from HI*. The core microbiome was determined at HI for both bleached and healthy corals at a 90% threshold, as this would capture taxa present across all samples (*n* = 5 per health status, per site, sensu Hernandez-Agreda et al. 2017). The majority of bacterial ASVs (healthy: 98.3% of ASVs, bleached: 99.3% of ASVs were not present across individuals with the same health status (Fig. 5A). In corals from HI, six ASVs were found in healthy samples and five ASVs were found in bleached samples. The core ASVs found associated with healthy samples were all in the phylum Proteobacteria, and included: an unidentified Gammaproteobacteria, two *Ralstonia spp*., a *Pelomonas spp.*, and two unidentified Oceanospirillales. The core ASVs found associated with bleached samples were all in the phylum Proteobacteria as well, and included: an *Alcanivorax spp*., a *Bradyrhizobium spp.*, two *Ralstonia spp*., and a *Pelomonas spp.* Of these, three ASVs were found in both healthy and bleached samples: two *Ralstonia spp*. and one *Pelomonas spp.* The presence of only three commonly identified taxa across all samples at 90% prevalence, as well as the majority of bacterial ASV's not found to be consistently present across at least 90% of samples, indicates that ASVs affiliated with rare bacterial taxa dominated the coral microbiome in the present study.

**Fig. 6:**
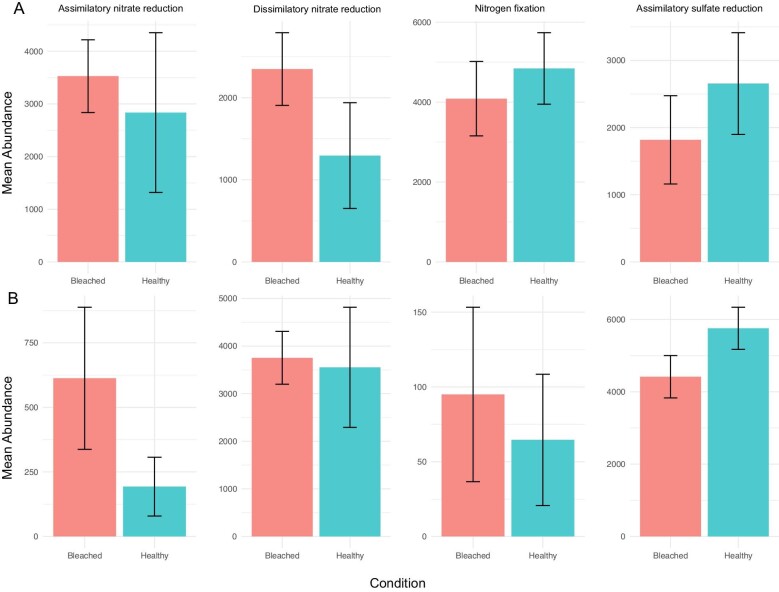
Barplot of mean abundance for key nitrate and sulfur metabolism pathways identified in consensus between PICRUSt2 and Tax4Fun2 between bleached and healthy corals at (**A**) HI and (**B**) LHI. No significant differences were observed between functions.

*Predicted microbial functional profiles do not differ between bleached and healthy corals at HI.* Herein focusing exclusively on nitrogen and sulphur metabolism, four KEGG pathways were identified in the microbial communities of bleached and healthy corals from HI: assimilatory nitrate reduction, dissimilatory nitrate reduction, nitrogen fixation, and assimilatory sulfate reduction. There was no clear enrichment of either nitrogen or sulphur metabolism KEGG pathways between healthy and bleached corals from HI (Fig. 6A).

### LHI coral bacterial communities

**Fig. 7:**
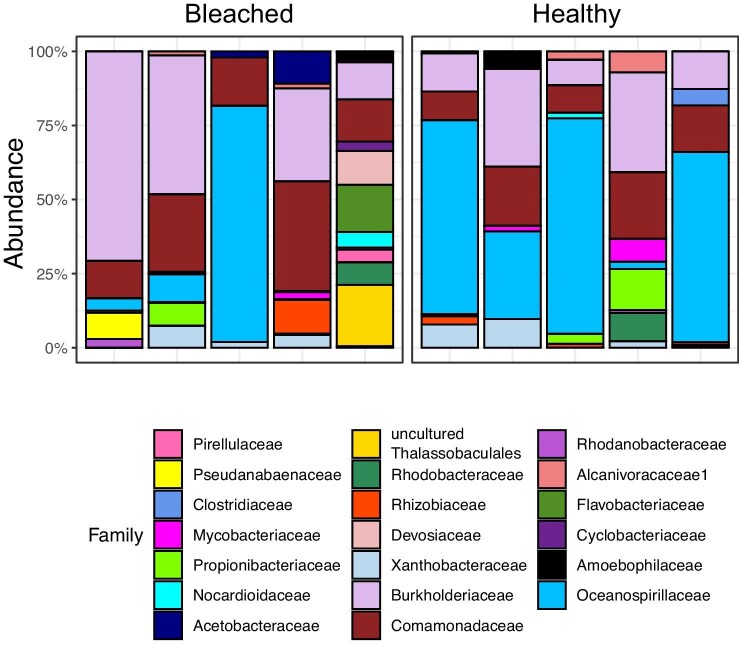
Relative % abundance of ASVs in the LHI community microbiome, agglomerated down to top 20 families and sorted by health status.

**Fig. 8:**
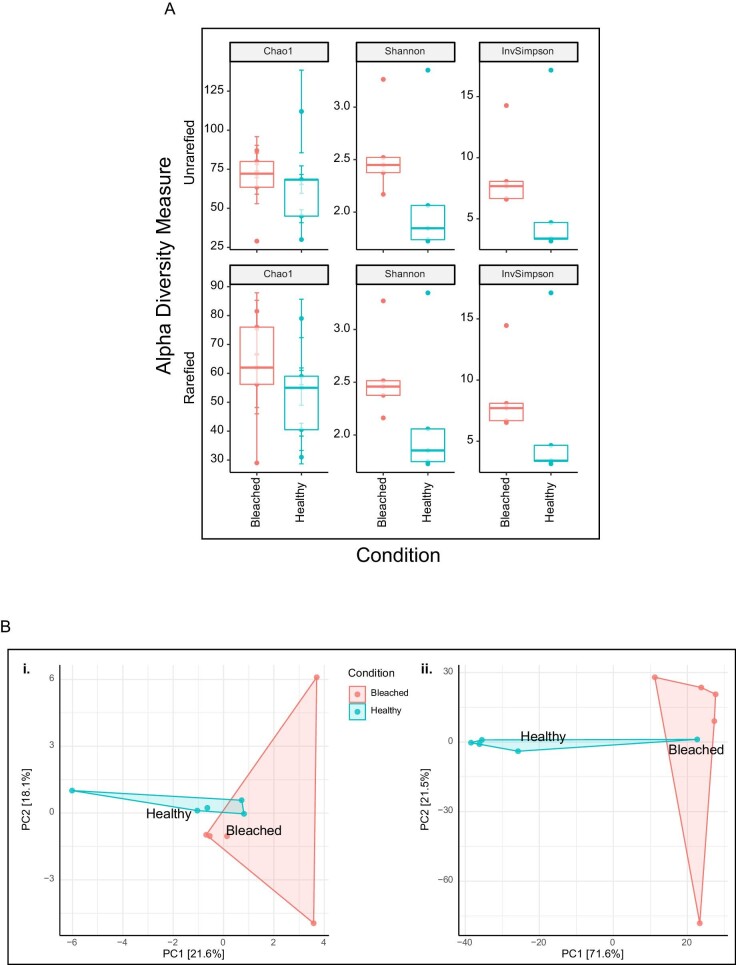
Community-level analyses for LHI. (**A**) Alpha diversity metrics on both unrarefied and rarefied data comparing healthy and bleached samples. Significant differences are indicated by an *. (**B**) Principal component analyses (PCoA) comparing clr-transformed (1) and rarefied (2) Euclidean distance matrices. No significant differences were observed in beta diversity metrics.

*Characteristics of the bleached coral microbiome from LHI.* Phylum-level assignment of bacteria ASVs indicated the dominance of Proteobacteria in both healthy (76% of taxa) and bleached (82% of taxa) samples. The most prevalent family in healthy samples from LHI was Comamonadaceae (9% of taxa), followed by Rhodobacteraceae (7% of taxa) and Beijerinckiaceae (5% of taxa) (Fig. 7). However, the families Oceanospirillaceae, Burkholderiaceae, and Propionibacteriaceae had the highest average relative abundances (Fig. 8A). Thalassospiraceae (11% of taxa) was the most prevalent family in bleached LHI samples, followed by Comamonadaceae (6% of taxa) and an uncultured bacterium in the order Thalassobaculales (5% of taxa). The families with the highest relative abundance in bleached LHI samples were Burkholderiaceae, Flavobacteriaceae, and Comamonadaceae (Fig. 7).

*No difference in alpha and beta diversity was found between the microbial communities of bleached and healthy samples from LHI.* There was no difference between healthy and bleached samples in Shannon (*F* = 1.307, *p* = 0.286), Chao1 (*F* = 0.008, *p* = 0.475), or Simpson (*F* = 0.561, *p* = 0.929) diversity metrics (Fig. 8A). Beta diversity did not differ between healthy or bleached bacterial communities of corals from LHI during the 2019 bleaching event (PERMANOVA on clr-transformed Euclidean distances, *F* = 1.157, *p* = 0.119; Fig. 8B).

*Differentially abundant ASVs in bleached and healthy coral samples from LHI do not include pathogenic taxa.* LeFSe analyses on LHI samples showed individual ASVs from the orders Oceanospirillales, Burkholderiales, and Pseudomonadales were higher in healthy samples. One ASV from the phylum Firmicutes was also higher in healthy samples. No members of the coral microbiome were higher in the bleached samples (Fig. 4B). Bleaching sensitivity is paralleled by the emergence of opportunistic bacterial species, but similarly to bleached samples from HI, there was no marked increase in relative abundance of *Vibrio* taxa. One Vibrionales species, *Photobacterium spp*., was present in one sample from LHI at <0.01% relative abundance, and no *Vibrio* taxa were present in bleached samples. Endozoicomonadaceae composed 3% of the taxa in bleached LHI samples, but this was driven by presence in a single sample at 0.002% relative abundance.

*Coral core microbiome in corals from LHI*. At a 90% threshold for core microbiome, 10 ASVs were found in healthy samples and 4 ASVs in bleached samples from LHI. The majority of bacterial ASVs were not present across individuals with the same health status (Fig. 5B). The core ASVs found associated with healthy samples were all in the phylum Proteobacteria, and included: two *Pelomonas spp.*, three unidentified Oceanspirillales, two *Ralstonia spp.*, two unidentified Gammaproteobacteria, and an uncultured *Oceanospirillum*. The core ASVs found associated with bleached samples were all in the phylum Proteobacteria as well, and included: two *Pelomonas spp*., an unidentified Gammaproteobacteria, and an uncultured *Oceanospirillum*. Of these, three ASVs were shared between both healthy and bleached samples: two *Pelomonas spp.* and an uncultured *Oceanospirillum.* At a 90% threshold, 94.6% of taxa were not present in healthy samples from LHI (188 taxa) and 98.1% of taxa were not present in bleached samples from LHI (213 taxa).

*Predicted microbial functional profiles do not differ between bleached and healthy corals at LHI.* Herein focusing exclusively on the fundamental properties of nitrogen and sulphur metabolism, four KEGG pathways were identified in the microbial communities of bleached and healthy corals from LHI: assimilatory nitrate reduction, dissimilatory nitrate reduction, nitrogen fixation, and assimilatory sulfate reduction. There was no clear enrichment of either nitrogen or sulphur metabolism KEGG pathways between healthy and bleached corals from LHI (Fig. 6).

## Discussion

In this study, we investigated the characteristics of the bleached *P. damicornis* coral microbiome during a reef wide bleaching event at two distinct coral reef locations, to determine the microbiome composition and inferred microbial functioning of the generalist coral. We found that stability in the coral microbiome, herein described as both (1) no change in beta diversity between healthy and bleached corals and (2) no increase in pathogenic taxa in bleached corals, was consistent in both coral reef lagoonal environments. Some taxa associated with both healthy and bleached corals at HI and LHI, such as Rhodobacteraceae and Flavobacteriia, were similar to what has previously been observed in *P. acuta* from Havannah Island and Pandora Reef on the GBR (Botté et al. 2022) and *P. damicornis* from HI (van Oppen et al. 2018). Ricci et al. (2022) found a high abundance of *Pseudoalteromonas* species in the tissues and skeleton of Pocilloporidae from the GBR that was not observed in the present study, possibly due to different bacterial communities associated with different sample collection methods targeting different regions of the holobiont (Bergman et al. 2022) or potentially seasonal difference in the microbiome which need to be assessed in future studies (Sharp et al. 2017; Yu et al. 2021b). To date, the *P. damicornis* bacterial microbiome at LHI has yet to be characterized, making this study the first to do so. In both locations, similar patterns in the microbial community in response to bleaching were observed.

Several patterns of microbial stability during bleaching emerged that were consistent in both distinct locations. These trends differ from two commonly presented microbial community hypotheses to heat stress. First, the AKP of dysbiosis, which suggests that microbial changes induced by perturbations lead to unstable community states and are stochastic (Zaneveld et al. 2017). Here, the stable microbiome composition observed (no significant difference between the microbiome of bleached and healthy corals from HI and LHI) is also similar to what has been reported during reef wide *P. acuta* thermal stress (Epstein et al. 2019) and *ex situ* simulated bleaching in *P. damicornis* (Bergman et al. 2021). Both Bergman et al. 2021 and Epstein et al. 2019 reported significant differences in beta diversity at the sequence variant level throughout *P. damicornis* thermal stress response (Epstein et al. 2019) and bleaching (Bergman et al. 2021), but further analysis revealed that no time points were driving this significance and led both studies to conclude overall bacterial stability in bleached colonies over time during a thermal stress event. Epstein et al. (2019) remark that in some coral species even significant thermal stress may not result in visible signs of bleaching; however, in both studies similar patterns of no marked increase in beta diversity was observed in the microbial communities of either bleached or heat stressed corals, and no clear pattern in beta diversity was identified to correlate to either bleaching or heat stress responses (Epstein et al. 2019; Bergman et al. 2021). Similarly, in the present study, variation in one metric of alpha diversity between healthy and bleached samples was identified (e.g., Shannon diversity in coral samples from HI), but no marked increases in beta diversity and Chao1/Simpson measures of alpha diversity were observed between healthy and bleached samples at both locations. Epstein et al. (2019) has shown that if heat stress does not result in severe bleaching or bleaching induced coral mortality, then the beta diversity of the coral microbiome is not altered. However, in the current study where large-scale coral bleaching and bleaching-induced mortality occurred across both reefs studied, community changes to the microbiome were not apparent at the time of sampling. These results may indicate that the timing of sampling within the bleaching event, reflective of the degree of heat stress and progression to mortality of the coral hosts, is potentially critical in understanding any correlation between bleaching, the microbiome, and bleaching outcomes for the coral colonies.

The complementary hypothesis to the AKP is a shift toward a pathogenic community similar to that found in diseased corals, which has been reported in heat-stressed corals (Bourne and Munn 2005; Thurber et al. 2009; Tout et al. 2015), but was also not found in the present study. *V. coralliilyticus*, a temperature-dependent pathogen of *P. damicornis* (Ben-Haim et al. 2003), and other *Vibrio* taxa were not found here. A lack of significant increase in *Vibrio*-affiliated sequences has also been observed in the *P. verrucosa* microbiome during a bleaching event in the South China Sea (Yang et al. 2021). Similar to Yang et al. (2021), the corals in the present study were collected during the reef wide bleaching event and outside of controlled aquaria conditions. Increases in the relative abundance of Rhodobacteraceae in bleached corals (as seen in the present study) has been associated with parallel increases in *Vibrio*-affiliated sequences previously in *P. damicornis* (Tout et al. 2015), but not in bleached *P.* *lutea* colonies (Pootakham et al. 2019) or in *P. damicornis* from HI undergoing thermal stress (Bergman et al. 2021). A caveat is that Pootakham et al. (2019) and Bergman et al. (2021), and the present study all used different reverse primers (519R or 1492R) to that used by Tout et al. 2015a) (1392R), so primer selection is also an important consideration.

Additionally, for corals collected from HI, the microbiome was almost entirely lacking *Endozoicomonas*, a common bacterial associate in Pocilloporid corals (Pogoreutz et al. 2018; Epstein et al. 2019; Voolstra and Ziegler 2020; Ricci et al. 2022), in both healthy and bleached/heat stressed samples. Ricci et al. (2022) characterized the microbiome of *P. damicornis* collected from HI at 0–1 m depth in January 2020, prior to bleaching events of 2020, and reported a high abundance of *Endozoicomonas* (uncultured species) associated with the coral tissues. The difference between our results and Ricci et al. (2022) suggests that warming water may disrupt the coral–*Endozoicomonas* association, an interpretation also supported by Botté et al. (2022) who reported low abundances of *Endozoicomonas* in *P. acuta* samples from Pandora Reef and Havannah Island on the GBR during a bleaching event and suggested that the onset of coral bleaching (3.5–5.6°C-weeks) represents a tipping point for *Endozoicomonas* species in *P. acuta*. As a loss of *Endozoicomonas* is often recorded in bleached or diseased corals (Bayer et al. 2013; Meyer et al. 2014; Glasl et al. 2016), an additional possibility suggested by Botté et al. (2022) is that repetitive and severe bleaching on the GBR has greatly reduced populations of coral tissue-associated *Endozoicomonas* over time. Epstein et al. (2019) found *Endozoicomonas* present in the majority of *P. acuta* samples from Orpheus Island collected in Feb—May 2016, prior to the repetitive mass bleaching events recorded from 2017, but found no variation in relative abundances between thermally stressed and healthy samples. As Epstein et al. (2019) also sampled corals during an *in situ* bleaching event, one interesting possibility is that all corals sampled in both Epstein et al. (2019) and the present study may have been environmentally stressed by reef wide warming regardless of bleaching status at the time of sample collection. This presents an alternative hypothesis to explain the lack of both *Endozoicomonas* and increased dysbiosis observed herein. In either instance, if *Endozoicomonas* populations do not return to their original abundances in a coral host following a bleaching event, then the coral host's resistance to stress may also decrease. However, further studies are needed to determine the cause of low abundances of *Endozoicomas* on the bacterial communities of corals following repetitive mass bleaching and sampled from the GBR in 2020, as in the present study.

In the current study, taxa that were found to be differentially abundant between bleached and healthy corals had varying functions. In healthy coral samples from HI, *Ulvibacter* spp. were found in a higher relative abundance. *Ulvibacter* spp. have been identified in seaweeds as a polysaccharide utilizer (Nedashkovskaya et al. 2016), which suggests that the organic carbon (e.g., cell wall of Symbiodiniaceae) in the coral holobiont may be utilized by *Ulvibacter* (Gong et al. 2020). In healthy corals, higher relative abundance of *Ulvibacter* could be aiding the coral holobiont in carbon uptake. More taxa were found to be at higher relative abundances in bleached corals than in healthy corals from HI, with individual ASVs from the families Rhodobacteraceae, Cyanobiaceae, Propionibacteriaceae, Ectothiorhodospiraceae, and Rhizobiaceae found at a higher abundance in bleached samples. Similar increases in the relative abundances of opportunistic Rhizobiales and Rhodobacteriales have been observed in *P. lutea* bacterial communities during an *in situ* bleaching event in the Andaman Sea (Pootakham et al. 2018), although similar to the results of the present study, no marked increase in relative abundances of disease-associated taxa (e.g., *Vibrio*) accompanied increases in Rhodobacteriales in heat-stressed corals (Pootakham et al. 2019). In healthy samples from LHI, individual ASVs from the orders Oceanospirillales and Burkholderiales were present in a higher abundance than in bleached samples, along with Pseudomonadales and the phylum Firmicutes. However, no ASVs were present at a higher abundance in bleached samples than healthy samples from LHI. A dominance of Oceanospirillales (47%) and Burkholderiales (8.5%) has previously been observed in many healthy coral species, including *P. damicornis* from HI (Tout et al. 2015; Ricci et al. 2022), in *Stylophora pistillata* from the Red Sea (Bayer et al. 2013), and *P. acuta* from Orpheus Island (Epstein et al. 2019). At LHI, both the healthy and bleached *P. damicornis* microbiome therefore show resemblance to a healthy coral microbiome, further supporting that no dysbiosis was observed in bleached *P. damicornis* samples.

At both HI and LHI, the majority of bacterial ASVs were not present across individuals with the same health status. One of the ASVs identified in both healthy and bleached samples from HI, as well as in healthy samples from LHI, belonged to *Ralstonia spp. Ralstonia* phylotypes have been commonly reported in studies of stony and soft corals (Sunagawa et al. 2010; Williams et al. 2015; Woo et al. 2017; Yu et al. 2021a). A *Pelomonas spp.* ASV was also observed in both healthy and bleached coral core microbiomes from both HI and LHI. *Pelomonas* is ubiquitous in coral microbiome studies (Röthig et al. 2017), but its role in the coral microbiome remains equivocal. It has been previously identified in the core microbiome of other corals such as the deep-sea coral *Eguchipsammia fistula* at a 100% threshold (Röthig et al. 2017) and *P. damicornis* at an 80% threshold (Bergman et al. 2021), removed as a contaminant (Pogoreutz et al. 2017), and associated with *A. hemprichii* samples from polluted sites (Ziegler et al. 2019). 1–6% of ASVs were shared across all members of a particular treatment group at either site, which is similar to a recent characterization of the *P. damicornis* microbiome that found 5–7% of all OTUs to be contained in the core microbiome (Ostria-Hernández et al. 2022). While one possibility is that widespread generalist species may not maintain a true coral microbiome (e.g., only one phylotype found persistent across three depth generalist coral species, Hernandez-Agreda et al. 2018b), the high specificity of coral core microbes also (Hernandez-Agreda et al. 2017, 2018b) suggests that variation in the abundance of the core microbes provides insight into the response of the coral host to stress. This is supported by Ostria-Hernández et al. (2022) finding that the relative abundance of the core microbiome of *P. damicornis* differed among anthropogenic stress levels. However, the core microbes that varied were not known coral pathogens (e.g., *Vibrio* species), which further supports microbial stability throughout bleaching in *P. damicornis*.

An important finding of the current study is that inferred functional profiles of the coral microbiome also did not differ between bleached and healthy corals at either location for the KEGG pathways of nitrogen and sulphur metabolism. Four functions were identified in bleached and healthy corals from HI and LHI: assimilatory nitrate reduction, dissimilatory nitrate reduction, nitrogen fixation, and assimilatory sulfate reduction. At both sites, there was no clear enrichment of either nitrogen or sulphur metabolism functions between healthy and bleached corals (Fig. 6). However, in interpreting these findings, acknowledging the resolution limitation of amplicon-based functional predictive tools is important as rare environment-specific functions may not be identified or maybe below the current detection limits, so these interpretations are made with caution (Douglas et al. 2020). A previous study comparing predicted functional profiles of the microbial communities of *P. damicornis* tissues and mucus reported 19 pathways to differ significantly between bleached and healthy microbial communities; however, similarly none of the altered functions were identified as nitrogen and sulfur metabolism (Zou et al. 2022). Additionally, for *P. verrucosa* during a bleaching event in the South China Sea, no difference in predicted functional profiles were found between bleached and healthy corals for nitrogen and sulfur metabolism pathways (Sun et al. 2022).

Overall, the characteristics of the bleached *P. damicornis* microbiome were similar in two distinct coral reef locations. Despite indications of bleaching, the bacterial communities and predicted functional roles of the bleached *P. damicornis* coral microbiome remained unchanged. It has been suggested that the role of *P. damicornis* and closely related members of its species complex (Schmidt-Roach et al. 2014) as environmental generalists may contribute to microbial stability throughout bleaching (Bergman et al. 2021), as similar stability has been observed in heat-stressed *P. damicornis* (Brener-Raffalli et al. 2018), *P. acuta* (Epstein et al. 2019; Botté et al. 2022), and *P. verrucosa* (Pogoreutz et al. 2018; Ziegler et al. 2019). Interestingly, a similar study comparing the microbiome of bleached and healthy coral species during a natural bleaching event in the Seychelles found stability was evident regardless of whether the coral species sampled was considered a “winner” or a “loser” in thermal tolerance (Gardner et al. 2019). This suggests that Symbiodiniaceae densities and bleaching response may be uncoupled from bacterial community, as seen in studies where the coral microbiome remains stable despite bleaching (Hadaidi et al. 2017). Overall, a conserved lack of differences in microbial community response to heat stress observed in the present study reflects a combination of host physiology and thermal stress responses that could be uncoupled from changes in the bacterial microbiome.

Finally, the conserved microbial response observed herein is also interesting given the different thermal regimes of the two bleaching events at HI and LHI. In a study of coral microbial assemblages from tropical and subtropical corals in the South China Sea, coral microbiome composition was found to vary across thermal regimes, with greater heterogeneity in corals from tropical reefs than subtropical possibly indicative of the microbial network transitioning from a stable to unstable state at bleaching threshold temperatures (Gong et al. 2020). In 2020, heat stress across the GBR (including HI) with max temperatures up to 37°C in 2020 (Ainsworth et al. 2021) highlighted the high temperatures experienced by the region on a cyclical basis. LHI has also experienced several bleaching events, documented in 2010, 2011, and 2019, with lower max temperatures of up to 25°C (Dalton et al. 2020; Steinberg et al. 2022). Additionally, seawater and sediments are one of the main drivers affecting microbiome composition, with more than 30% of ASVs shared between surrounding seawater, sediment, and *P. damicornis* on the GBR at a point in time (Ricci et al. 2022). Different seawater and sediment microbial communities are likely to differ between sites and therefore contribute to the differences in microbial community seen between sites herein. Future studies should therefore also examine seawater and sediment microbial communities, in order to understand if the taxa observed are site-specific to the coral, sediment, or seawater environment.

Natural variation between factors in corals *in situ* may also contribute to masking changes in the coral microbiome and are addressed here. For example, in the present study, different colonies on the reef flat were sampled at each timepoint. This contrasts with Epstein et al. (2019), who sampled the same colony throughout thermal stress events. While Epstein et al. (2019) found no change in the bacterial microbiome of the same colonies over time, it's possible that in the present study the microbiome of a single colony may have changed following bleaching but that natural variability is masked by colony-specific variation. In addition, natural variation in time since onset of bleaching (the caveat of an *in situ* experiment vs. a controlled *ex situ* experiment) could be an important factor explaining the breadth of variation observed amongst the bleached colonies, which would also mask any bleaching-specific variation. This raises the question that while we recorded photophysiological decline and symbiont loss concomitant with bleaching in the present study, it's not possible to know the length of time since the onset of bleaching or if corals were eventually pushed to mortality *in situ*. Both of these would affect the eventual development of dysbiosis in the coral microbiome and contribute to the variation within groups observed in the present study. With the small sample size in the present study, variation within groups could also potentially mask differences in diversity or composition among groups. We did not detect significant differences between groups in the present study, but future studies not limited by sample size could have increased statistical power to disentangle variability within groups from variability among groups. These factors are all caveats that should be considered when conducting an experiment *in situ* and in the interpretation of the results herein.

In conclusion, by examining the microbial communities of healthy and bleached corals during reef wide bleaching events of two distinct coral reef locations, we find consistent bleaching responses of the bacterial community between tropical (HI) and subtropical (LHI) Pocilloporid corals. While differences in the bacterial taxa of the holobiont confirm other studies reporting distinct bacterial communities associated with coral reef habitats (Hernandez-Agreda et al. 2016), an overall consistently stable microbial community response (e.g., no increase in diversity or shift toward a dysbiotic state) suggests that there is the possibility of a generalized host-specific or environment-specific influence on the holobiont of *P. damicornis* bleaching responses. Microbial community stability conserved across environments may contribute to the role of *P. damicornis* as a widespread environmental generalist species. Structurally stable microbiomes, presumed to be strongly selected, may host bacterial communities with a specialized set of functions (Ley et al. 2006; McDevitt-Irwin et al. 2017; Ziegler et al. 2017). It has been suggested that stable members play important roles in coral health, whereas transient members may vary with environmental conditions or perturbances (McDevitt-Irwin et al. 2017; Hernandez-Agreda et al. 2018b). Our results highlight the need for the inclusion of a broad range of (1) sites with varying thermal regimes and (2) species with different functional traits (e.g., specialist vs. generalist) to comprehensively characterize key members of a bleached and healthy coral microbiome.

## Supplementary Material

obad012_Supplemental_File

## Data Availability

The sequencing data underlying this article are available in an NCBI repository accessible at https://www.ncbi.nlm.nih.gov/, PRJNA802894.
