# Supplementary material for: Characteristics of The Bleached Microbiome of The Generalist Coral Pocillopora damicornis from Two Distinct Reef Habitats
Source: Integr Org Biol. 2023 Apr 28;5(1):obad012. doi: 10.1093/iob/obad012 (PMC10084919; doi:10.1093/iob/obad012)
Supplement: obad012_Supplemental_File [file obad012_supplemental_file.docx]

**Supplementary Data**

Table S1 – Reads per sample and site, including negative controls.

| Sample Name | Site | Condition | Reads |
| --- | --- | --- | --- |
| JB14 | LHI | Healthy | 33218 |
| JB15 | LHI | Healthy | 10468 |
| JB16 | LHI | Healthy | 26682 |
| JB17 | LHI | Healthy | 19420 |
| JB18 | LHI | Healthy | 21261 |
| JB19 | LHI | Bleached | 19174 |
| JB20 | LHI | Bleached | 26079 |
| JB21 | LHI | Bleached | 11329 |
| JB22 | LHI | Bleached | 10089 |
| JB23 | LHI | Bleached | 11368 |
| JB2 | HIRS | Healthy | 20413 |
| JB3 | HIRS | Healthy | 815 |
| JB4 | HIRS | Healthy | 41209 |
| JB5 | HIRS | Healthy | 1623 |
| JB6 | HIRS | Healthy | 3913 |
| JB7 | HIRS | Bleached | 6284 |
| JB8 | HIRS | Bleached | 15908 |
| JB9 | HIRS | Bleached | 15434 |
| JB10 | HIRS | Bleached | 8343 |
| JB11 | HIRS | Bleached | 1648 |
| NEG1 | HIRS | N/C | 201 |
| NEG2 | HIRS | N/C | 2600 |
| NEG3 | LHI | N/C | 1575 |
| NEG4 | LHI | N/C | 2654 |

Table S2 – taxa removed from samples based on a decontam threshold of 0.5

| **ASV** | **Site** | **Kingdom** | **Phylum** | **Class** | **Order** | **Family** | **Genus** | **Species** |
| --- | --- | --- | --- | --- | --- | --- | --- | --- |
| ASV1 | HIRS | Bacteria | Proteobacteria | Alphaproteobacteria | Rhizobiales | Xanthobacteraceae | Bradyrhizobium | Bradyrhizobium_sp. |
| ASV2 | HIRS | Bacteria | Proteobacteria | Alphaproteobacteria | Acetobacterales | Acetobacteraceae | Acidocella | NA |
| ASV3 | HIRS | Bacteria | Actinobacteriota | Actinobacteria | Propionibacteriales | Propionibacteriaceae | Cutibacterium | uncultured_bacterium |
| ASV4 | HIRS | Bacteria | Proteobacteria | Gammaproteobacteria | Oceanospirillales | Alcanivoracaceae1 | Alcanivorax | uncultured_Alcanivorax |
| ASV5 | HIRS | Bacteria | Proteobacteria | Alphaproteobacteria | Rhizobiales | Rhizobiaceae | Mesorhizobium | Rhizobium_sp. |
| ASV6 | HIRS | Bacteria | Bacteroidota | Bacteroidia | Chitinophagales | Chitinophagaceae | Hydrobacter | NA |
| ASV7 | HIRS | Bacteria | Proteobacteria | Alphaproteobacteria | Rhizobiales | Xanthobacteraceae | Bradyrhizobium | Bradyrhizobium_sp. |
| ASV8 | HIRS | Bacteria | Proteobacteria | Gammaproteobacteria | Burkholderiales | Hydrogenophilaceae | Hydrogenophilus | Hydrogenophilus_hirschii |
| ASV9 | HIRS | Bacteria | Proteobacteria | Gammaproteobacteria | Burkholderiales | Comamonadaceae | Pelomonas | NA |
| ASV10 | HIRS | Bacteria | Proteobacteria | Gammaproteobacteria | NA | NA | NA | NA |
| ASV11 | HIRS | Bacteria | Proteobacteria | Gammaproteobacteria | Burkholderiales | Comamonadaceae | Pelomonas | NA |
| ASV2 | LHI | Bacteria | Proteobacteria | Alphaproteobacteria | Acetobacterales | Acetobacteraceae | Acidocella | NA |
| ASV12 | LHI | Bacteria | Actinobacteriota | Actinobacteria | Propionibacteriales | Propionibacteriaceae | Cutibacterium | uncultured_bacterium |
| ASV13 | LHI | Bacteria | Proteobacteria | Alphaproteobacteria | Rhodospirillales | Thalassospiraceae | Thalassospira | NA |
| ASV14 | LHI | Bacteria | Actinobacteriota | Actinobacteria | Corynebacteriales | Mycobacteriaceae | Mycobacterium | uncultured_organism |
| ASV15 | LHI | Bacteria | Proteobacteria | Alphaproteobacteria | Sphingomonadales | Sphingomonadaceae | Porphyrobacter | NA |
| ASV16 | LHI | Bacteria | Proteobacteria | Alphaproteobacteria | Rhizobiales | Xanthobacteraceae | Bradyrhizobium | Bradyrhizobium_sp. |
| ASV17 | LHI | Bacteria | Actinobacteriota | Actinobacteria | Corynebacteriales | Mycobacteriaceae | Mycobacterium | uncultured_organism |
| ASV18 | LHI | Bacteria | Proteobacteria | Gammaproteobacteria | Burkholderiales | Rhodocyclaceae | Dechlorobacter | uncultured_anaerobic |
| ASV19 | LHI | Bacteria | Proteobacteria | Alphaproteobacteria | Acetobacterales | Acetobacteraceae | Acidocella | NA |
| ASV20 | LHI | Bacteria | Proteobacteria | Gammaproteobacteria | Burkholderiales | Burkholderiaceae | Pandoraea | Burkholderia_sp. |
